# Affirming educational and workplace settings are associated with positive mental health and happiness outcomes for LGBTQA + youth in Australia

**DOI:** 10.1186/s12889-023-16034-7

**Published:** 2023-07-25

**Authors:** Natalie Amos, Adam O. Hill, Jami Jones, G. J. Melendez-Torres, Marina Carman, Anthony Lyons, Adam Bourne

**Affiliations:** 1 Australian Research Centre in Sex, Health and Society, La Trobe University, Building NR6, Bundoora, VIC 3086 Australia; 2 Graduate School of Public Health, St Luke’s International University, Tokyo, Japan; 3 College of Medicine and Health, University of Exeter, Exeter, England UK; 4 Kirby Institute, UNSW Sydney, Wallace Wurth Building, High Street, Kensington, NSW 2052 Australia

**Keywords:** LGBTQA, Affirmation, Education, Work, Youth, Mental health, Wellbeing, Happiness

## Abstract

**Background:**

Affirming socio-cultural settings are essential for protecting the mental health and wellbeing of lesbian, bisexual or pansexual, trans and gender diverse, asexual and queer (LGBTQA +) youth. However, limited research has explored the role of affirming educational and workplace settings, as reported by LGBTQA + youth themselves, with respect to their mental health and wellbeing. Moreover, existing research maintains a focus on mitigating poor mental health outcomes, with little attention to positive wellbeing outcomes among LGBTQA + youth.

**Methods:**

Using data from the largest national survey of LGBTQA + youth aged 14–21 in Australia, multivariable regression analyses were conducted to explore associations between affirming educational and workplace settings and psychological distress and subjective wellbeing among 4,331 cisgender and 1,537 trans and gender diverse youth. Additionally, a series of multivariable regression analyses were conducted to explore individual sociodemographic traits that are associated with reporting affirming educational or workplace settings.

**Results:**

Both cisgender and trans or gender diverse participants who reported that their education institution or workplace were affirming of their LGBTQA + identity reported lower levels of psychological distress as well as higher levels of subjective happiness. Additionally, affirming environments were not experienced equally across all subsections of LGBTQA + youth, with reporting of an affirming educational or workplace setting differing most noticeably across gender, type of educational institution and residential location.

**Conclusion:**

The findings demonstrate that affirming educational and workplace settings can result not only in better mental health, but also greater levels of subjective happiness among LGBTQA + youth. The outcomes illustrate the importance of ensuring all LGBTQA + youth are afforded the opportunity to thrive in environments where they feel validated and confident to express their identities. The findings further highlight a need to target education institutions and workplaces to ensure the implementation of policies and practices that promote not just inclusion of LGBTQA + youth but affirmation of their identities.

## Background

Research among lesbian, bisexual or pansexual, trans and gender diverse, asexual and queer (LGBTQA +) youth, both globally and in Australia, consistently illustrate disproportionately poor mental health outcomes among sexual and gender diverse young people compared to their non-LGBTQA + peers [1–5]. LGBTQA + young people are not inherently mentally unwell and existing research has explored a myriad of factors, external to the individual, that likely contribute to mental health concerns, such as experiences of discrimination, harassment, rejection of their LGBTQA + identity from family and others, or attempts to change their identity through conversion practices [1, 3, 6]. Evidently, harmful interpersonal interactions and un-affirming cultural and societal reactions to a young person’s gender or sexual identity can play a detrimental role in their mental wellbeing.

Conversely, affirming experiences are likely to protect mental health and result in positive wellbeing outcomes [3]. However, limited research has explored the role of socio-cultural settings indicated by LGBTQA + youth themselves to be affirming of their identity. Outside of family, LGBTQA + youth’s interactions with others and broader socio-cultural settings are most likely to occur in an education setting or workplace. Consequently, these settings are important to target for research and interventions to ensure affirming environments for young LGBTQA + people, while also offering an opportunistic setting for public health initiatives.

Increasingly, research has explored the role of inclusive practices and policies within educational settings for the mental health of LGBTQA + youth. Several factors within educational environments have been identified that contribute to experiences of safety and inclusivity. These include, for example, anti-bullying policies, support groups such as gay-straight alliances, professional development for faculty that relates to LGBTQA + student issues, and the inclusion of LGBTQA + identities in the curriculum [7–10]. While these policies and practices are designed to create inclusive and safe environments for LGBTQA + youth and may lead to experiences of affirmation [9], an affirmative approach goes beyond inclusivity and safety to create an environment that recognises, validates and supports the identity stated or expressed by LGBTQA + youth [11, 12].

Evidence of LGBTQA + -inclusive education environments are associated with numerous positive health and wellbeing outcomes for sexual and gender minority students, such as decreased intimate partner violence among female students [7], better mental health outcomes and less suicide-related behaviours [9, 13–15], decreased experiences of bullying or harassment [8, 9, 15], increased connection or sense of belonging at school [9], and less illicit drug use [9, 15]. Furthermore, a recent Australian survey of school experiences among LGBTQA + young people aged 13–18 years found that students who reported that their school had harassment policies that named sexual orientation as a protected category had higher average wellbeing scores [10].

These studies evidence the importance of school policies and practices in creating inclusive environments that may be experienced as affirming for LGBTQA + young people and highlight the specific approaches that may contribute to a positive environment. However, few studies have explored how feelings of affirmation within an education environment as expressed by young people themselves, impacts their wellbeing. Additionally, the existing education-based research predominantly focusses on school settings, with limited research exploring post-secondary institutions, such as universities and vocational education settings (i.e., technical and further education institutions).

Less research has focused on workplace settings (as compared to educational settings), particularly among young people. None-the-less, the limited research that does exist provides compelling evidence of the significance a workplace environment may have for the wellbeing of LGBTQA + youth. LGBTQA + people are more likely than their cisgender and heterosexual colleagues to experience discrimination and harassment in the workplace [16, 17] at an interpersonal and organisational level [18]. As is the case in educational settings, policies and practices within workplaces have been identified that result in better outcomes for LGBTQA + people. These include, for example, workplace diversity training and workplace employee networks or ally networks [19]. LGBTQ people within workplaces that have inclusive policies and practices in place, are more likely to feel that their workplace is affirming or safe [19, 20] and to feel safe to disclose their identity [20, 21], as well as experiencing greater equality and better career prospects within the workplace [22]. However, little research has explored the mental health or wellbeing implications of affirming workplaces for LGBTQA + people, and none to our knowledge with a focus on LGBTQA + youth.

One study of LGBTQA + veterinary professionals and students based in the US and UK found that evidence of an affirming workplace or study environment was associated with better mental health and wellbeing outcomes [23]. These outcomes, while specific to veterinarians and combining both students and professionals, suggest the significant role that an affirming workplace climate is likely to play for the wellbeing of LGBTQA + youth. Further research is necessary to directly explore these associations, particularly with regard to young people and their experience of affirming environments within the workplace.

Notably, experiences of educational and workplace settings may differ across subsections of the LGBTQA + youth population. For example, trans and gender diverse individuals report even higher rates of discrimination within schools than cisgender sexual minority youth [1, 24] and workplaces [17, 19], while also reporting less inclusion in school curricular and less discussion of gender diversity from teachers [10]. Furthermore, experiences of affirmation may differ between those who are cisgender sexual minorities, and those who are trans or gender diverse. For example, trans and gender diverse young people are likely to be more impacted by their ability to comfortably access bathrooms within an education setting, while cisgender sexually diverse young people may be more impacted by their ability to comfortably engage in public affection with a person of the same gender [25]. Additionally, cisgender men are likely to experience greater career opportunities and higher pay than women in the workplace [26]. However, limited research has directly explored which subsections of LGBTQA + youth are most or least likely to experience affirming environments. This knowledge may highlight where biases in inclusive or affirming policies and practices exist within educational or workplace settings.

Moreover, the existing literature focuses predominantly on negative wellbeing outcomes, such as mental health and suicidality [9, 13, 23], with little attention paid to positive wellbeing outcomes, such as happiness. The prevention of mental health concerns among young people should be considered a bare minimum. Beyond the absence of poor mental health, research, policy and practice must aspire for the positive wellbeing of all young people. Therefore, the present study aims to explore the role of affirming educational and workplace environments on the psychological distress as well as subjective happiness of LGBTQA + youth. As discussed above, trans and gender diverse youth are likely to experience affirmation in differing ways to cisgender sexual minority youth, therefore the relationships explored will be examined separately for cisgender youth and trans and gender diverse youth.

This study seeks to determine whether cisgender sexually diverse youth and trans or gender diverse youth who indicate that their education or workplace settings are affirming of their identity experience lower levels of psychological distress and greater levels of happiness. In addition, the study will use an exploratory approach to identify who is most or least likely to report an affirming educational or workplace setting, exploring the sociodemographic factors that are associated with these experiences. The outcomes of this paper will provide knowledge that is essential for informing policy and practice efforts in the primary prevention of poor mental health outcomes and fostering of positive wellbeing, particularly with regard to the role of affirming environments.

## Method

### Sample and procedure

The study sample involved data from the *Writing Themselves In 4* national survey of the health and wellbeing of 6,418 LGBTQA + young people aged 14–21 years in Australia [1]. The survey was open for completion in late 2019, and participants were recruited via targeted social media advertising as well as promotion by LGBTIQ community organisations. Participants were provided with a Plain Language Statement detailing the study protocol and overview of the survey. They were then asked to indicate their consent to participate online prior to starting the survey. Consent was not obtained from parents or guardians for younger participants. This is in acknowledgement of the fact that many young people may have not disclosed their gender identity or sexuality to their parents or guardians. Indeed, doing so could result in harm for those in circumstances where their parents/guardians are not supportive of such identities or experiences. All survey questions, beyond eligibility criteria, were optional. Ethics approval was obtained from the La Trobe University Human Research Ethics Committee. The present paper analyses the data of 4,331 cisgender and 1,537 trans and gender diverse participants who responded to questions regarding their educational setting environment, and 2,869 cisgender and 658 trans and gender diverse participants who responded to questions regarding their workplace environment.

### Materials

#### Demographics

Demographic variables included age (categorised into 14–17 years and 18–21 years); sexual orientation (gay/lesbian, bisexual, pansexual, queer, asexual or something else); gender (cisgender man, cisgender woman, trans men, trans woman, non-binary or other gender diverse term); area of residence (inner-suburban, outer-suburban, regional city or town, rural or remote); county of birth (Australian born, another English-speaking country; a non-English speaking country); and educational setting (high school, university, TAFE [vocational education aged over 16 years], other).

#### Psychological distress

Psychological distress was assessed using the Kessler Psychological Distress Scale (K10) The K10 is a ten-item standardised scale designed to measure level of psychosocial distress in the past four weeks and has been validated among young people in Australia [27]. Each item asks about experiences relating to symptoms of stress, psychological fatigue and depression, with a 5-point Likert response ranging from 1 (“None of the time”) to 5 (“All of the time”). Scores are computed by summing responses to each of the items and can range from 10–50, with scores of 22 or more indicating high or very high level of psychological distress according to the Australian Bureau of Statistics guidelines [28].

#### Subjective happiness

Subjective happiness was measured using the 4-item self-report internationally validated Subjective Happiness Scale (SHS) [29, 30]. This is a widely used scale which reliably determines an individual’s subjective experience of happiness. This scale was chosen as it is not tied to a specific life domain but rather provides a global measure of happiness. Participants respond to items using a 7-point Likert scale, such as “In general I consider myself…” with responses ranging from 1 “Not a very happy person” to 7 “A very happy person”. Following reverse coding of one negatively worded item, responses to all 4 items are averaged. Scores range from 1–7, with higher scores indicating greater happiness [30].

#### Affirming educational and workplace settings

Scales were developed for the purpose of this study to assess participants’ experiences of affirming workplace and educational settings. These items were developed for the purpose of the *Writing Themselves In 4* survey in collaboration with a Community Advisory Board and Youth Advisory Group. Due to unique affirming experiences between cisgender sexual minority young people and trans or gender diverse young people that may not apply to both groups, such as using chosen name or pronouns, different scales were used to determine affirming experiences for cisgender and trans or gender diverse young people. These scales are described in greater detail below.

##### Affirming educational setting – cisgender young people

To determine whether participants’ educational setting was experienced as affirming of their LGBTQA + identity, a score was computed using a set of items that assessed whether or not participants felt that they could comfortably identify or present as LGBTQA + within their educational setting. Participants were asked “During the past 12 months at your education institution have you felt that you could safely…”. Participants were then asked to select as many of the following items that applied to them: “Engage in public affection (PDA) with other LGBTIQA + people”; “Attend a school dance with someone of the same gender”; “Openly identify as LGBTIQA + ”; “Celebrate ‘Wear it Purple day’, IDAHOBIT, or Transgender Day of Visibility or another LGBTIQA + day of significance.” Participants were also given the option to select “None of the above”. Response to these items were then coded with a 1 “Yes” or a 0 “No”. Responses were then summed together to create a scale score ranging from 0–4, with a higher score indicating a more affirming experience. A tetrachoric matrix was conducted to determine the internal reliability of this scale revealing *r* = 0.4–0.7, suggesting good interitem correlations with an alpha of 0.7.

##### Affirming educational setting – trans and gender diverse young people

Trans and gender diverse young people were asked additional questions relating to their experience of comfort and safety within the school setting with the same format as described above. These additional items included “Use the bathrooms/changing rooms that match my gender identity”, “Use my chosen name or pronouns” and “Wear clothes that match my gender identity.” Again, participants could indicate “None of the above”. Response to these items, plus “Openly identify as LGBTIQA + ” and “Celebrate ‘Wear it Purple day’, IDAHOBIT, or Transgender Day of Visibility or another LGBTIQA + day of significance” as described above for cisgender participants, were summed to form a total score for an affirming educational setting specific to trans and gender diverse young people. Scores ranged from 0–5, with higher scores indicating a more affirming experience. A tetrachoric matrix was conducted to determine the internal reliability of this scale revealing *r* = 0.3–0.7, suggesting good interitem correlations with an alpha of 0.7.

##### Affirming workplace – cisgender young people

Similarly, to education setting, to determine whether participants’ workplace was affirming of their LGBTQA + identity, a score was computed using a set of items that assessed whether or not participants felt that they could comfortably identify or present as LGBTQA + within their workplace. Participants were asked “During the past 12 months at your place of work have you felt that you could safely…”. Participants were then asked to select as many of the following items that applied to them: “Engage public affection (PDA) with other LGBTIQA + people”, “Openly identify as LGBTIQA + ” and “Celebrate ‘Wear it Purple day’, IDAHOBIT, or Transgender Day of Visibility or another LGBTIQA + day of significance.” Participants were also given the option to select “None of the above”. Response to these items were then coded with a 1 “Yes” or a 0 “No”. Responses were then summed together to create a scale score ranging from 0–3, with a higher score indicating a more affirming experience. A tetrachoric matrix was conducted to determine the internal reliability of this scale revealing *r* = 0.6–0.8, suggesting good interitem correlations with an alpha of 0.7.

##### Affirming workplace – trans and gender diverse young people

Trans and gender diverse young people were asked additional questions relating to their experience of comfort and safety within the workplace with the same format as described above. These additional items included: “Use the bathrooms/changing rooms that match my gender identity”, “Use my chosen name or pronouns” and “Wear clothes that match my gender identity.” Again, participants could indicate “None of the above”. Response to these items, plus “Openly identify as LGBTIQA + ” and “Celebrate ‘Wear it Purple day’, IDAHOBIT, or Transgender Day of Visibility or another LGBTIQA + day of significance” as described above for cisgender participants were summed to form a total score for an affirming workplace specific to trans and gender diverse young people. Scores ranged from 0–5, with higher scores indicating a more affirming experience. A tetrachoric matrix revealed *r* = 0.4–0.7, suggesting good interitem correlations with an alpha of 0.7.

### Statistical analyses

All analyses were performed using STATA (Version 16.1, StataCorp, College Station, TX, USA). In order to explore whether affirming workplace and educational settings were associated with psychological distress or subjective happiness scores, a series of multivariable regression analyses were conducted with psychological distress and happiness scores as the outcome variables. Affirming educational setting and affirming workplace were included in separate models as predictor variables. In addition, each model controlled for sociodemographic variables including gender, sexual orientation, level of education, country of, and residential location. Separate models were run among those who identified as cisgender, and those who identified as trans or gender diverse.

A series of multivariable regression analyses were also conducted to explore the sociodemographic variables that were associated with reporting an affirming education or workplace environment. The scale scores for affirming education and workplace environments were the outcome variables, with sociodemographic variables as described above, included in the model as predictor variables. These were again run as separate models among those who identified as cisgender, and those who identified as trans or gender diverse.

Tests of multicollinearity indicated that this was not a concern for any of the regression analyses conducted, with all Variance Inflation Factors (VIFs) < 2. Results are reported as Beta coefficients (β) with 95% confidence intervals (CIs) and *P* < 0.05 used to assess statistical significance.

## Results

In total, 4,331 cisgender young people and 1,537 trans and gender diverse young people responded to questions about their education setting. Additionally, 2,869 cisgender young people and 658 trans and gender diverse young people responded to the workplace environment questions. Frequencies and proportions of sample characteristics are reported in Table 1, these are divided into those who responded to questions about their workplace environment, and those who responded to the educational environment questions among cisgender and trans and gender diverse participants.

**Table 1:** Sample characteristics

	**Education setting**				**Workplace**			
	**Cisgender**		**Trans or gender diverse**		**Cisgender**		**Trans or gender diverse**	
	**n**	**%**	**n**	**%**	**n**	**%**	**n**	**%**
**Gender—cisgender**								
Cisgender woman	3027	69.9	-	-	1945	67.8	-	-
Cisgender man	1304	30.1	**-**	-	924	32.2	**-**	-
**Gender—trans or gender diverse**								
Trans woman	-	-	67	4.4	-	-	21	3.2
Trans man	-	-	367	23.9	-	-	145	22.0
Non-binary	-	-	1103	71.8	-	-	492	74.8
**Sexual orientation**								
Lesbian/gay	1415	32.7	277	18.0	961	33.5	109	16.6
Bisexual	1702	39.4	333	21.7	1130	39.4	144	21.9
Pansexual	324	7.5	299	19.5	205	7.2	104	15.8
Queer	241	5.6	242	15.7	176	6.1	129	19.6
Asexual	152	3.5	94	6.1	100	3.5	47	7.1
Something else	488	11.3	292	19.0	293	10.2	125	19.0
**Education**								
Secondary school (high school)	2792	64.5	912	59.3	1509	55.3	293	49.3
University	1129	26.1	365	23.7	955	35.0	203	34.2
TAFE	216	5.0	143	9.3	149	5.5	60	10.1
Other	194	4.5	117	7.6	115	4.2	38	6.4
**Country of birth**								
Australia born	3813	88.3	1384	90.2	2545	88.9	589	89.6
Other English-speaking country	271	6.3	89	5.8	179	6.3	50	7.6
Non-English-speaking country	235	5.4	61	4.0	139	4.9	18	2.7
**Residential location**								
Capital city, inner suburban	299	6.9	91	5.9	244	8.5	46	7.0
Capital city, outer suburban	2587	59.7	839	54.7	1695	59.1	352	53.7
Regional city or town	1019	23.5	419	27.3	650	22.7	178	27.1
Rural/Remote	425	9.8	185	12.1	279	9.7	80	12.2

### Affirming educational and workplace settings and mental wellbeing

Table 2 presents results of the regression analysis exploring the association between affirming workplace and education environments among those who are cisgender and those who are trans or gender diverse. These regression analyses controlled for the effects of several sociodemographic factors including gender, sexual orientation, education level, country of birth (Australian born; born in another English-speaking country; born in a non-English speaking country), and residential location (inner-suburban; outer-suburban; regional city or town; rural or remote area).

**Table 2:** Associations between affirming environments and wellbeing among cisgender and trans or gender diverse youth

	**Cisgender**		**Trans or gender diverse**	
	**B (95% CI)**	***P*-Value**	**B (95% CI)**	***P*-Value**
**Education setting**^**a**^				
Psychological distress^b^	-0.99 (-1.18—-0.80)	0.000	-0.91 (-1.19—-0.63)	0.000
Subjective happiness^b^	0.17 (0.14—0.21)	0.000	0.15 (0.11—0.19)	0.000
**Workplace**^**a**^				
Psychological distress^b^	-0.77 (-1.08—-0.46)	0.000	-0.71 (-1.12—-0.30)	0.001
Subjective happiness^b^	0.16 (0.11—0.22)	0.000	0.07 (0.00—0.14)	0.047

All regression analyses controlled for sociodemographic factors including age, gender, sexual orientation, education, country of birth and residential location

^a^ Predictor variable

^b^ Outcome variable

#### Educational setting

Among both cisgender sexual minority young people and trans or gender diverse young people an affirming educational environment was associated with lower levels of psychological distress (cisgender: β = -0.99, CI = -1.18–0.8, *p* < 0.001; trans or gender diverse: β = -0.91, CI = -1.19–0.63, < 0.001) and higher levels of happiness (cisgender: β = 0.17, CI = 0.14–0.21, *p* < 0.001; trans or gender diverse: β = 0.15, CI = 0.11–0.19, *p* < 0.001).

#### Workplace

Similarly, among both cisgender sexual minority young people and trans or gender diverse young people an affirming workplace environment was associated with lower levels of psychological distress (cisgender: β = -0.77, CI = -1.08–0.46, *p* < 0.001; trans or gender diverse: (β = -0.71, CI = -1.12–0.3, *p* = 0.001) and higher levels of happiness (cisgender: β = 0.16, CI = 0.11–0.22, *p* < 0.001; trans or gender diverse: β = 0.07, CI = 0–0.14, *p* = 0.047).

### Individual characteristics associated with reporting affirming educational or workplace settings

Table 3 presents outcomes from regression analyses exploring the sociodemographic factors that are associated with reporting affirming workplaces and education settings among LGBTQA + young people, divided into cisgender young people and trans and gender diverse young people.

**Table 3:** Correlates of affirming educational or workplace environment among cisgender and trans or gender diverse youth

	**Educational setting**				**Workplace**			
	**Cisgender**		**Trans or gender diverse**		**Cisgender**		**Trans or gender diverse**	
	**B (95% CI)**	***P*-Value**	**B (95% CI)**	***P*-Value**	**B (95% CI)**	***P*-Value**	**B (95% CI)**	***P*-Value**
**Gender—cisgender**								
Cisgender woman	REF		-	-	REF		-	-
Cisgender man	-0.10 (-0.19 – 0.00)	0.045	-	-	0.23 (0.14—0.33)	0.000	-	-
**Gender—trans or gender diverse**								
Trans woman	-	-	REF		-	-	REF	
Trans man	-	-	0.68 (0.25—1.11)	0.002	-	-	1.48 (0.57—2.40)	0.001
Non-binary	-	-	0.58 (0.17—0.98)	0.006	-	-	1.02 (0.15—1.89)	0.022
**Sexual orientation**								
Lesbian/gay	REF							
Bisexual	-0.02 (-0.12—0.08)	0.749	0.10 (-0.14—0.34)	0.414	0.01 (-0.09—0.11)	0.791	-0.23 (-0.68—0.21)	0.302
Pansexual	0.17 (0.01—0.34)	0.042	-0.29 (-0.54—-0.03)	0.030	0.07 (-0.10—0.25)	0.410	-0.49 (-0.97 – 0.00)	0.048
Queer	-0.05 (-0.23—0.13)	0.608	0.15 (-0.11—0.40)	0.257	-0.01 (-0.19—0.16)	0.882	-0.20 (-0.65—0.24)	0.371
Asexual	-0.10 (-0.32—0.13)	0.386	-0.21 (-0.56—0.14)	0.250	-0.07 (-0.30—0.15)	0.530	-0.92 (-1.51—-0.34)	0.002
Something else	-0.11 (-0.25—0.03)	0.119	0.11 (-0.15—0.37)	0.402	-0.10 (-0.24—0.05)	0.193	-0.04 (-0.50—0.41)	0.846
**Education**								
Secondary school (high school)	REF							
University	0.24 (0.16—0.33)	0.000	1.22 (1.04—1.40)	0.000	-0.02 (-0.11—0.07)	0.714	0.21 (-0.09—0.52)	0.172
TAFE	-0.01 (-0.18—0.15)	0.869	0.77 (0.50—1.04)	0.000	0.04 (-0.14—0.23)	0.638	0.35 (-0.15—0.86)	0.168
Other	-0.06 (-0.26—0.13)	0.542	0.68 (0.36 – 1.00)	0.000	0.03 (-0.17—0.22)	0.785	-0.17 (-0.76—0.42)	0.571
**Country of birth**								
Australia born	REF							
Other English-speaking country	0.04 (-0.13—0.2)	0.658	0.17 (-0.14—0.48)	0.275	0.01 (-0.15—0.18)	0.866	-0.37 (-0.88—0.14)	0.151
Non-English-speaking country	-0.09 (-0.27—0.09)	0.339	-0.04 (-0.40—0.33)	0.838	-0.19 (-0.37—-0.01)	0.041	0.53 (-0.28—1.34)	0.199
**Residential location**								
Capital city, inner suburban	REF							
Capital city, outer suburban	-0.25 (-0.4—-0.10)	0.001	-0.40 (-0.73—-0.08)	0.015	-0.22 (-0.37—-0.07)	0.004	-0.31 (-0.82—0.20)	0.238
Regional city or town	-0.33 (-0.50—-0.16)	0.000	-0.35 (-0.7 – 0.00)	0.047	-0.36 (-0.53—-0.19)	0.000	-0.16 (-0.71—0.40)	0.580
Rural/Remote	-0.48 (-0.68—-0.28)	0.000	-0.79 (-1.17—-0.4)	0.000	-0.29 (-0.49—-0.09)	0.004	-0.52 (-1.17—0.12)	0.110

#### Educational setting

Cisgender participants were less likely to report an affirming educational setting if they were cisgender men (β = -0.1, CI = -0.19–0, *p* = 0.045) and more likely if they identified as pansexual, as compared to gay or lesbian (β = 0.17, CI = 0.01–0.34, *p* = 0.042). Cisgender participants were also more likely to report an affirming education environment if they attended a university (β = 0.24, CI = 0.16–0.33, *p* < 0.001). Cisgender participants were less likely to report an affirming educational setting if they resided outside of inner-suburban areas, with those least likely to report an affirming education setting residing in a rural or remote area (outer-suburban area: β = -0.25, CI = -0.4–0.1, *p* = 0.001; regional city or town: β = -0.33, CI = -0.5–0.16, *p* < 0.001; rural or remote area: β = -0.48, CI = -0.68–0.28, *p* < 0.001).

Trans and gender diverse young people were more likely to report an affirming educational setting if they were trans men (β = 0.68, CI = 0.25–1.11, *p* = 0.002) or non-binary (β = 0.58, CI = 0.17–0.98, *p* = 0.006), as compared to trans women, and less likely to report and affirming educational setting if they identified as pansexual (β = -0.29, CI = -0.54–0.03, *p* = 0.030). Additionally, participants who attended educational environments other than secondary school were all more likely to report affirming settings, with those attending university most likely to report an affirming educational setting (University: β = 1.22, CI = 1.04–1.4, *p* < 0.001; TAFE: β = 0.77, CI = 0.5–1.04, *p* < 0.001; other educational setting: β = 0.68, CI = 0.36–1, *p* < 0.001). Similarly, to cisgender participants, trans and gender diverse young people were less likely to report an affirming educational setting if they lived outside of inner-suburban areas, with those living in rural or remote areas the least likely to report an affirming educational setting (outer-suburban area: β = -0.4, CI = -0.73–0.08, *p* = 0.015; regional city or town: β = -0.35, CI = -0.7–0, *p* = 0.047; rural or remote area: β = -0.79, CI = -1.17–0.4, *p* < 0.001).

#### Workplace

Among cisgender young people, participants were most likely to report affirming workplaces if they were cisgender men (β = 0.23, CI = 0.14–0.33, *p* = 0), and less likely to report affirming workplaces if they were born in an a non-English speaking country (β = -0.19, CI = -0.37–0.01, *p* = 0.041), and if they lived outside of inner-suburban areas, with those least likely to report affirming workplaces residing in a regional city or town (outer-suburban area: β = -0.22, CI = -0.37–0.07, *p* = 0.004; regional city or town: β = -0.36, CI = -0.53–0.19, *p* < 0.001; rural or remote area: β = -0.29, CI = -0.49–0.09, *p* = 0.004).

Among trans and gender diverse young people, only gender and sexual orientation were associated with reporting an affirming workplace. Participants were most likely to report affirming workplaces if they were trans men (β = 1.48, CI = 0.57–2.4, *p* = 0.001) or non-binary (β = 1.02, CI = 0.15–1.89, *p* = 0.022), and less likely if they identified as pansexual (β = -0.49, CI = -0.97–0, *p* = 0.048). Additionally, those who identified as asexual were less likely to report an affirming workplace environment (β = -0.92, CI = -1.51–0.34, *p* = 0.002). However, this finding is likely due to a small sample size, with only 54 trans and gender diverse participants who answered the workplace environment questions identifying as asexual.

## Discussion

There are several known contributors to the poor mental health of LGBTQA + young people that exist outside of the individual [1, 3, 6]. While a necessary focus has been to identify those young people at greatest risk of mental health concerns and ensure that appropriate mental healthcare is available to them, an increased focus is needed on the prevention of mental health concerns in the first instance. It is evident that LGBTQA + -affirming experiences are important for protecting the mental health of LGBTQA + people [3]. Among both cisgender and trans and gender diverse youth reporting that their workplace or their education institution was affirming of their LGBTQA + identity (i.e., they felt safe to disclose, identify or express their identity in the workplace or their education institution) was associated with lower levels of psychological distress and higher levels of subjective happiness. Additionally, affirming environments were not experienced equally across all subsections of the population, with differences most noticeably evident across gender, type of educational institution and residential location.

### Educational setting

LGBTQA + youth showed lower levels of psychological distress and greater levels of subjective happiness the more affirmed they felt in their educational setting. Contrary to the approach taken in the present study, previous research has predominantly explored the role of educational environments by examining evidence of LGBTQA + -inclusive policies and practices ([9], e.g., [13, 15]), as opposed to exploring young people’s experiences of affirming environments. However, evidence from existing literature suggests that there is likely to be a strong association between inclusive policy and practice and experiences of affirmation among young people [9]. Moreover, the existing literature has focused principally on associations with poor mental health outcomes [9, 13–15]. Importantly, the present study illustrates that not only are experiences of affirming educational environments associated with lower levels of psychological distress, experiences of affirmation in an educational environment can also foster greater levels of happiness.

Finding that cisgender men were less likely to report an affirming school environment as compared to cisgender women may relate to how presentations of masculinity and femininity are valued within the school setting and broader society [31]. Previous research suggests that heterosexual men perceive masculine gay men more positively than feminine gay men [32] and suggests greater discomfort with homosexuality among heterosexual men stemming from a perceived threat that gay men pose to traditional ideas of Western masculinity [33]. Accordingly, cisgender men experience higher rates of harassment based on their sexuality within an educational setting than do cisgender women [1]. It is unsurprising then that they are less likely to feel affirmed within their education institutions.

Finding that cisgender participants who identified as pansexual were the most likely to report an affirming educational setting was not anticipated, particularly given the higher rates of poor mental health reported by young people who identify as pansexual as compared to those with a different sexual orientation [1]. One possible explanation is that young people who identify as pansexual are more likely to already be in an affirming educational setting. Given the recency of awareness and language for pansexual identities [34, 35], many young people may not have been afforded an opportunity to explore this identity. However, young people in already affirming environments may have increased access to discourse and language for diverse sexual identities, enabling them to express and identify in this way. This is only a speculative explanation for this finding and qualitative research is needed to explore the expression and experiences of affirmation among young people of diverse sexual identities.

Furthermore, cisgender participants who attended universities being more likely than those attending secondary school to report that their educational setting was affirming may reflect more progressive views and greater indicators of inclusivity toward LGBTQA + identities that have previously been found within university settings [1, 36].

Finally, cisgender participants living outside of inner-suburban areas were all less likely to feel that their educational setting was affirming of their identity. These results may reflect the political or cultural climate of these areas of residence as compared to inner-suburban areas. Inner-suburban areas are generally more progressive in their political views than outer areas [37, 38], and therefore may be more LGTBQA-affirming [39]. A study of LGBTQ adolescents in Canada found that adolescents who lived in areas where a higher percentile of residents vote for the progressive political party experienced better mental health outcomes, suggesting that these areas are more LGBTQ-friendly [40]. The political views of these areas may shape the workplace and educational environments in these regions, and consequently young people’s experiences of affirmation in these settings.

Among young people who identified as trans or gender diverse, trans men and non-binary young people were more likely than trans women to report an affirming educational environment. This outcome may, again, relate to how presentations of masculinity and femininity are valued within these settings [33]. It may be easier for trans men and non-binary individuals to present as trans-masculine without drawing as much criticism or abuse for these identities and expressions as masculine presentations may be valued higher in educational settings and receive less ridicule than feminine presentations [31, 32]. From an intersectionality perspective, these findings underscore the complex interplay of gender identity and societal expectations and biases related to femininity and womanhood. Trans women may face unique challenges as a consequence of transmisogyny, a societal prejudice at the intersection of transphobia [41, 42] and misogyny resulting in less affirming environments as indicated by young trans women. Accordingly, trans women have been shown to report higher rates of harassment based on sexual orientation or gender identity within educational settings [1].

Converse to the findings for cisgender youth, pansexual trans or gender diverse young people were the least likely of those who were trans or gender diverse to report an affirming educational environment. It is unclear why the findings for sexual orientation differ between the two gender groups and may reflect complexities arising from intersecting identities. Further research exploring in-depth how affirmation is experienced across LGBTQA + identities and intersecting traits is needed.

Similar to the findings among cisgender young people, trans and gender diverse young people were more likely to report affirming educational settings if they attended a university, TAFE, or other educational setting, compared to secondary school, and less likely to report affirming educational settings if they lived outside of inner-suburban areas. Again, these outcomes likely reflect the more progressive views within post-secondary institutions and inner-suburban areas, as discussed above.

### Workplace

Reporting an affirming workplace among LGBTQA + young people was also associated with lower psychological distress and greater subjective happiness. Previous research exploring the role of affirming workplace environments and wellbeing is very limited, and there are no studies to our knowledge that focus specifically on young LGBTQA + people. As with respect to educational settings, research in workplaces looks predominantly at evidence of inclusive policies and practices, rather than the experience of employees themselves. One study that explored the role of affirming workplace climates, as reported by employees, was conducted in the US and UK among veterinarians [23]. While this study combined both student and professional veterinarians, their results reflect those of the present study and suggest that an affirming workplace environment was associated with better mental health outcomes [23]. Crucially, the present study explores beyond the mitigation of poor mental health (as has been the focus of existing literature) and illustrates that not only can an affirming workplace protect mental health, it can also result in greater happiness among young LGBTQA + employees.

Among cisgender participants, contrary to the education setting findings, cisgender men were the most likely to feel that their workplace was affirming of their identity, likely reflecting ongoing advantages in workplace environments afforded to cisgender men [43]. Cisgender participants were less likely to report an affirming workplace environment if they were born in a non-English speaking country, likely reflecting additional discriminations experienced by participants of culturally and linguistically diverse backgrounds [26]. Finally, similar to educational settings, cisgender participants living outside of inner-suburban areas were all less likely to feel that their workplace was affirming of their identity. As discussed with regard to educational settings above, this outcome likely reflects more progressive views held within inner-suburban areas.

Among trans and gender diverse people, sexual orientation was the only sociodemographic trait associated with experiencing an affirming workplace environment, with asexual people least likely to report affirming workplace environments. However, this finding is likely due to the small sample size in this group of participants, as detailed in the results section. Finding no other associations with experiencing an affirming workplace may suggest that trans and gender diverse youth generally do not feel affirmed within workplaces and that their gender alone outweighs the impact of other intersecting identities or traits.

### Limitations and future research

The present study allowed for a detailed observation of the role of affirming educational and workplace settings in the mental health and wellbeing outcomes of LGBTQA + youth. However, this study is not without its limitations. While the scales used to assess affirming workplace and educational settings demonstrated good internal validity, these were not standardised measures. The items were created in collaboration with a Community Advisory Board (CAB) and are not dissimilar to measures used in previous literature to explore LGBTQ issues. Moreover, the scales included items understood by the investigator and CAB members to be central to an affirming experience, but there are likely other important factors to consider at the interpersonal and structural level of these settings relating to experiences of affirmation. Further qualitative research is necessary to explore the interactions and environmental forces that shape affirming experiences for LGBTQA + young people. Additionally, as the study relies on cross-sectional survey data, causality cannot be presumed from the findings. However, attempts were made to align the timeline of experiences in both school and workplace settings with the reported wellbeing outcomes. This enables a more robust interpretation of the relationships between affirming environments and wellbeing, even within the limitations of a cross-sectional study design. Finally, it is a major strength of this study that the overall survey sample was large enough to be able to disaggregate analyses into nuanced categories, particularly regarding multiple gender identities and sexual orientations. However, the sample size of those who responded to workplace questions is more modest and when broken down by sociodemographic categories in the regression analyses, the individual samples become relatively small. Future research with a specific focus on work environments and LGBTQA + youth may benefit from targeted recruitment to gain a larger sample size. Finally, it may be of interest, and political importance, for future research to focus on quality-of-life outcomes for LGBTQA + youth in relation to affirming environments as a means of further quantifying the importance of these affirming environments and reduction in mental healthcare needs.

## Conclusion

Experiencing affirming educational and workplace settings likely plays a critical role in the mental health and wellbeing of young LGTBQA+ people in Australia. The mitigation of poor mental health is the bare minimum that LGBTQA + young people should expect. Crucially, this study shows that affirming environments can also result in greater happiness among LGBTQA + youth and demonstrates an opportunity within educational and workplace settings to do so. The outcomes of this study highlight the importance of ensuring all LGBTQA + youth are afforded the opportunity to thrive in environments where they feel safe and comfortable to disclose and express their identities. Legislature and public health initiatives targeting education institutions and workplaces are required to ensure the implementation of policies and practices that promote not just inclusion of LGBTQA + youth but affirmation of their identities. Outcomes from the present study additionally illustrate a need for these approaches to specifically target educational and workplace settings in residential locations outside of inner-city areas.

## Data Availability

The datasets generated and/or analysed during the current study are not publicly available for the privacy and protection of participants but are available from the corresponding author on reasonable request.

## Abbreviations

LGBTQA +: Lesbian, gay, bisexual or pansexual, trans and gender diverse, asexual and queer
K10: Kessler Psychological Distress Scale
